# Recruitment of IFT proteins during flagellum construction in *Trypanosoma brucei*

**DOI:** 10.1186/2046-2530-4-S1-P47

**Published:** 2015-07-13

**Authors:** E Bertiaux, P Bastin, B Rotureau

**Affiliations:** 1 Trypanosome Cell Biology Unit, INSTITUT PASTEUR, Paris, France

## Introduction

*Trypanosoma brucei *is a protozoan parasite responsible for sleeping sickness in Central Africa. It is transmitted by the bite of the tsetse fly. During its complex life cycle, it undergoes significant morphological changes including extensive variations in flagellum length (3 to 30 µm). The flagellum is an essential organelle for parasite survival as it is involved in morphogenesis, movement, division and adhesion of the trypanosome. Intraflagellar transport (IFT) refers to the movement of protein complexes between the membrane and the microtubules. Like in other eukaryotes, IFT is essential for the construction of the trypanosome flagellum. Studies in the green alga *Chlamydomonas *suggested that the total amount of IFT proteins injected in the flagellum defines its final length: the higher the amount, the longer the flagellum. Moreover, the total amount of IFT proteins would be injected at once.

## Methods

We have studied the distribution of IFT proteins during the development of *Trypanosoma brucei*. Expression and localization of IFT proteins was analyzed by immunofluorescence with antibodies against two IFT proteins (IFT22 and IFT172).

## Results

The analysis of fluorescence intensities revealed that the total amount of IFT proteins in the flagellum is directly proportional to its length in all stages examined, as observed in *Chlamydomonas*. However, the IFT protein concentration per unit of flagellum length is constant at any steps of flagellum formation. These results were confirmed by monitoring protein trafficking in live cells expressing the TdTomato::IFT81 fusion protein.

## Conclusions

Our results lead to a new model where IFT proteins would be progressively recruited to the flagellar compartment during elongation of the organelle. This raises the question of the regulation of IFT injection to the flagellum that will be addressed by studying the formation of very long or very short flagella in several development stages in the tsetse fly.

